# Niche differentiation rather than biogeography shapes the diversity and composition of microbiome of *Cycas panzhihuaensis*

**DOI:** 10.1186/s40168-019-0770-y

**Published:** 2019-12-02

**Authors:** Ying Zheng, Xun Gong

**Affiliations:** 1 Key Laboratory for Plant Diversity and Biogeography of East Asia, Kunming Institute of Botany, Chinese Academy of Sciences, Kunming, Yunnan China; 2 Key Laboratory of Economic Plants and Biotechnology, Kunming Institute of Botany, Chinese Academy of Sciences, Kunming, Yunnan China; 3 University of Chinese Academy of Sciences, Beijing, China

**Keywords:** Microbiome, Niche differentiation, Biogeography, Diversity, Plant compartment, Transmission model

## Abstract

**Background:**

Given their adaptation to nutrient-poor and drought environments, cycads are vital models for plant-microbiome interaction research because they are likely to host an important reservoir of beneficial microbes that may support cycad survival. However, a comprehensive understanding of the diversity and community composition of microbiome associated with different plant compartments as well as bulk soils of cycad species remains elusive.

**Method:**

An extensive investigation of species diversity and community composition of bacterial and fungal microbiome in roots, seeds, unfertilized seeds, ovules, pollens, and soils of *Cycas panzhihuaensis* L. Zhou & S. Y. Yang has been conducted by high-through sequencing technology. Moreover, principal component analysis (PCA), hierarchical cluster analysis (HCA), and heatmap analysis were applied to test the niche-specific effect and biogeography factor among different sample types of this cycad species.

**Results:**

Highly diverse microbiota and significant variation of community structure were found among different compartments of *C*. *panzhihuaensis*. Soils exhibited a remarkable differentiation of bacterial community composition compared to the other five plant organs as revealed by PCA, HCA, and heatmap analyses. Different compartments possessed unique core microbial taxa with Pseudomonadaceae and Nectriaceae shared among them. According to the indicator species analysis, there was almost no differentiation of dominant microbiomes with regard to the geography of the host cycad. Two main transmission models existed in the *C*. *panzhihuaensis*.

**Conclusions:**

Each sample type represented a unique niche and hosted a niche-specific core microbial taxa. Contrary to previous surveys, biogeography hardly exerted impact on microbial community variation in this study. The majority of the cycad-associated microbes were horizontally derived from soils and/or air environments with the rest vertically inherited from maternal plants via seeds. This study offers a robust knowledge of plant-microbiome interaction across various plant compartments and soils and lends guidelines to the investigation of adaptation mechanism of cycads in arid and nutrient-poor environments as well as their evolutionary conservation.

## Background

Being the host’s second and extended genome, plant microbiome hosts taxonomically diverse microbes, including bacteria, fungi, protists, and viruses. Great attentions have been paid to this entity as it contributes to plant fitness and productivity by providing a plethora of functional capacities such as access to low-abundance nutrients, suppression of phytopathogens, and resistance to biotic and/or abiotic stressors. Three categories have been classified according to the interaction between host plants and microbiota: negative (pathogenic) interaction, positive interaction, and neutral interaction [1, 2]. Among those interactions, symbiosis has been considered as synergistic interaction benefiting both partners. The microbes provide various essential nutrients (i.e., nitrogen and phosphorous) to the host species, which promotes plant growth and increases environmental tolerance and phytopathogenic resistance. In return, the plant partners offer stable niches and photosynthetic productions to the microbiota.

Almost every plant taxon is colonized by a highly diverse and finely structured microbe communities [3]. Each plant compartment serves as a unique ecological niche for microbial entities, hosting a distinct microbial assembly as compared to other plant tissues, including roots, stems, leaves, flowers, and seeds [4–8]. In addition, soil microbiota represents a common species reservoir for plant-associated microbiomes. The majority of these microorganisms were supposed to be recruited from soil environments [9, 10] via the following few steps. Soil-borne microbiota firstly migrated to the rhizosphere and concentrated around the rhizoplane of the host, which was induced by root exudates and rhizodepositions. After host selection, some rhizoplane-dwelling microbes penetrated the host roots and gradually colonized the interior tissues (namely horizontal transmission). However, the microbial colonization process does not complete at this step. Further transmission to the above-ground parts of the host, i.e., stems, flowers, and seeds, continued (namely vertical transmission, coupled with a more general pathway via seeds to the next generation). Horizontal dissemination through air-plant interfaces such as the phyllosphere has also been reported. Noticeably, the transmission process is far from perfect. Only certain microbial assembly is capable to colonize the aerial part of plants, primarily due to the physical barriers (i.e., strengthened cell wall and gum inside vessels) and immune system of the host, as well as the incapability of some microbial taxa to adapt to plant internal microenvironments [7]. In most plants, more microbes are found in below-ground tissues than in above-ground parts [11, 12]. Beckers et al. described a higher diversity for the rhizosphere soil of field-grown poplar (*Populus tremula* × *Populus alba*) than the samples of endosphere plant compartments [13]. Furthermore, the microbial community compositions of stem and leaf samples were clearly distinguished from rhizosphere soil and root samples, and core bacterial microbiome associated with different niche of poplar were uncovered. Structural variability and niche differentiation were proposed to reshape the diversity and composition of microbiome of this model species [13].

Cycads are ancient and contemporary relic gymnosperm. They are dioecious and mainly entomophilous pollination [14, 15]. Some species thrive in stressful environment characterized by arid and nutrient-poor conditions, such as *Cycas panzhihuaensis* L. Zhou & S. Y. Yang. The distribution of *C*. *panzhihuaensis* is restricted to dry-hot valleys of the Jinsha River in southwest China [16]. Due to the foehn effect and topographical enclosure, the weather in the valleys of the Jinsha River is dominated by high temperature and low humidity with shallow soils and fragile geological structure [17]. It has been supposed that the evolutionary conservation and survival in nutrient-poor and harsh environmental conditions of cycads is linked with the ability to form symbioses with various microbial consortia, especially with cyanobacteria in the specialized apogeotropic roots for nitrogen acquisition [18, 19]. The occurrence and colonization process of cyanobacteria into coralloid roots of cycads [20, 21], as well as their diversity and community compositions [18, 22–25] have been well-documented. For instance, high cyanobacterial diversity in coralloid roots of cycads was revealed by PCR fingerprinting [25]. Contrarily, Costa and Lindblad argued that only a single *Nostoc* strain was presented in individual coralloid roots of cycads by using the tRNA^Leu^ (UAA) intron as a genetic marker [22]. More recently, comparative analysis of endophytic microbes between coralloid roots and regular roots of *Cycas bifida* via the high-through sequencing techniques has been reported by Zheng et al. uncovering a highly diverse endophytic microbiome in roots of cycad [26]. Given their adaption to nutrient-poor and drought environments, cycads are vital models for plant microbiome research, which may preserve important resources of beneficial microbes that may support their survival. However, studies disentangling the microbiome of most cycad species and the comparison of microbial community among different tissues of cycad plant have rarely been conducted. Likewise, the origin and transmission mode of cycads microbiota remained elusive.

The rapid development and wide application of next-generation sequencing (NGS) techniques in metagenomics has uncovered numerous genetic codes of plant microbiomes, regardless of their culturability [27]. Benefiting from its high read quantity and quality, the Illumina MiSeq sequencing platform has been widely applied to microbial community analysis [28–30]. Previous studies by culture and isolation of strains in *C*. *panzhihuaensis* have revealed diverse endophytic fungi in this cycad species, and similar microbial community was detected between samples from natural and cultural habitats. The variation of fungal community composition among different plant tissues was obvious [31]. Some strains are un-culturable and the exact diversity and abundance of endophytes in *C*. *panzhihuaensis* may be underestimated. In this study, using the Illumina MiSeq sequencing platform, seeds, unfertilized seeds, pollens, ovules, roots, and bulk soils of *C*. *panzhihuaensis* were sampled from natural and managed habitats to test the following three hypotheses:

I. Niche differentiation among different sample types influenced the diversity and composition of cycad-associated microbiome.
II. The biogeography of the host cycad shaped the community structure of microbiome.
III. Two different sources of microbe existed in *C*. *panzhihuaensis* with the majority of them derived horizontally from soil.

## Results

### Quality metrics of high-through sequencing analysis

A total of 3,800,658 raw reads, 1,816,516 from 16S rRNA, and 1,984,142 from ITS were identified among 32 samples of *C*. *panzhihuaensis* prior to quality control (QC) and assignation (Table 1). For sequences detected by the 16S rRNA gene marker, the mean raw read length before QC was 395.04 bp. After quality trimming and assigning reads to different samples, 1,156,635 high-quality reads were remained in the 16S rRNA dataset with an average read length of 377.09 bp. Among them, 1,036,039 reads were mapped to Greengenes and retrieved 1,036,034 Bacteria reads. No archaeal and mitochondrial sequences were co-amplified during the sequencing process. A small fractions of chloroplast reads were co-amplified from unfertilized seeds (1), ovules (2), and roots (2). The highest number of bacterial read was identified in samples of unfertilized seeds (52,235.33), followed by ovule samples (47,944.75). Soils contained the least bacterial read number (19,637.75 for soil samples from KM and 15,772 from PZH). Cyanobacteria was the most dominant bacteria in coralloid roots of cycads. In this research, 292 cyanobacterial reads were identified in *C*. *panzhihuaensis*. Most of them were derived from the coralloid root sample: 219 from KM and 55 from PZH. Cyanobacteria were detected in all the samples collected from natural habitats of *C*. *panzhihuaensis*, yet no cyanobacteria was detected in seeds, unfertilized seeds, ovules, or soil samples from its cultivated habitat.

**Table 1:** Summary of sequencing reads from all barcodes and samples

		KM						PZH		
	Assignation	Seed	Unfertilized seed	Ovule	Pollen	Root	Soil	Seed	Root	Soil
16S	Total	52030	63796.33	58669.50	60436.25	40875.50	62932	60753	58110.25	54228
	Raw read length before QC	394.40	395.32	394.93	394.60	395.33	395.61	394.08	395.31	395.56
	Read length after QC	376.32	377.33	376.95	376.61	377.50	377.61	376.08	377.59	377.56
	Processed to haplotypes	31177	53781.33	49629	38547.75	30146	22399	38232.67	42342.50	18268.33
	Map to Greengenes OTU	26055.67	52235.67	47945.25	32134.75	27498.25	19637.75	32823	36629	15772
	Bacteria	26055.67	52235.33	47944.75	32134.75	27498	19637.75	32823	36628.75	15772
	Cyanobacteria	0	0	0	3	54.75	0	1.67	13.75	0.33
	Chloroplast	0	0.33	0.50	0	0.25	0	0	0.25	0
ITS	Total	57993.33	61671.67	61446.50	58594	61563.50	74690.50	59343	55869.25	66154.33
	Raw read length before QC	252.88	260.31	245.79	246.81	245.07	242.91	247.97	239.58	240.22
	Read length after QC	236.72	242.16	227.75	227.88	225.65	224.21	231.27	220.88	220.78
	Processed to haplotypes	47333.33	51395.67	52772.75	50316.75	54629.25	66946.25	47152	47834	62131
	Map to Genebank OTU	21203	17878.33	25299	23424.75	17628.50	37209.75	15838.67	26968.50	39869.33
	Fungi	21202.67	17878.33	25299	23422.25	17625	37209.75	15827.67	26968.50	39869.33
	Plantae	0.33	0	0	2.50	3.50	0	11	0	0

The number represented the mean value of each sample type

For ITS sequences, the mean raw read length before QC was 246.40 bp, and 228.07 bp after QC (Table 1). Further, 1,714,032 high-quality ITS sequences were processed to haplotypes, resulting in 806,432 fungal reads, and 58 Plantae reads. In contrast to 16S rRNA results, samples of soil contained the most fungal read number (37,209.75 for KM samples and 39,869.33 for PZH) and seeds from PZH contained the least (15,827.67). Fifty-eight reads were assigned to Plantae, identifying seven OTUs, three from seeds, two from pollens, and two from roots.

After assembling and assigning high-quality reads to each dataset, Greengenes Database for bacteria (August 2013 release; http://greengenes.lbl.gov) and UNITE v7.0 for fungi at a 97% sequence similarity level, 728,240 bacterial haplotypes and 145,221 fungal haplotypes were retrieved with a total of 13,907 bacterial operational taxonomical units (OTU) and 3320 fungal OTUs generated (Table 2). Although soil samples contained the least bacterial read number, it was assigned to the highest OTU number (1219.5 for KM and 897.67 for PZH) compared to the other five sample types. Ovules contained the least bacterial OTUs (91.75). Twelve cyanobacterial OTUs were identified. Each of the seed (PZH), pollen (KM), and soil (PZH) compartments contained one cyanobacteria OTU. Coralloid roots from KM included three cyanobacteria OTUs, and coralloid roots from PZH included six. For fungi, the most OTU was detected in sample ovules (176.5) and the least was roots from KM (69.5).

**Table 2:** Summary of haplotypes and operational taxonomical unit (OTU) numbers detected using two markers

		KM						PZH		
	Assignation	Seed	Unfertilized seed	Ovule	Pollen	Root	Soil	Seed	Root	Soil
16S	Haplotypes	23609.67	11352	9714	24117	12096	44112.75	24980.67	17957.50	38808
	Total OTU	237.33	147.33	92	322.25	285.75	1219.50	334.33	345.75	897.67
	Bacteria	237.33	147	91.75	322.25	285.50	1219.50	334.33	345.50	897.67
	Cyanobacteria	0	0	0	0.25	0.75	0	0.33	1.50	0.33
	Chloroplast	0	0.33	0.25	0	0.25	0	0	0.25	0
ITS	Haplotypes	2750.67	2820	6217.25	3747.50	2559	7917.25	2886	3102.75	8558.67
	Total OTU	58.67	83	176.50	108.75	36.50	161	107.33	103	79
	Fungi	58.33	83	176.50	108.25	36	161	106.67	103	79
	Plantae	0.33	0	0	0.50	0.50	0	0.67	0	0

The number represented the mean value of each sample type

### Rarefaction curves and diversity indices

**Fig. 1:**
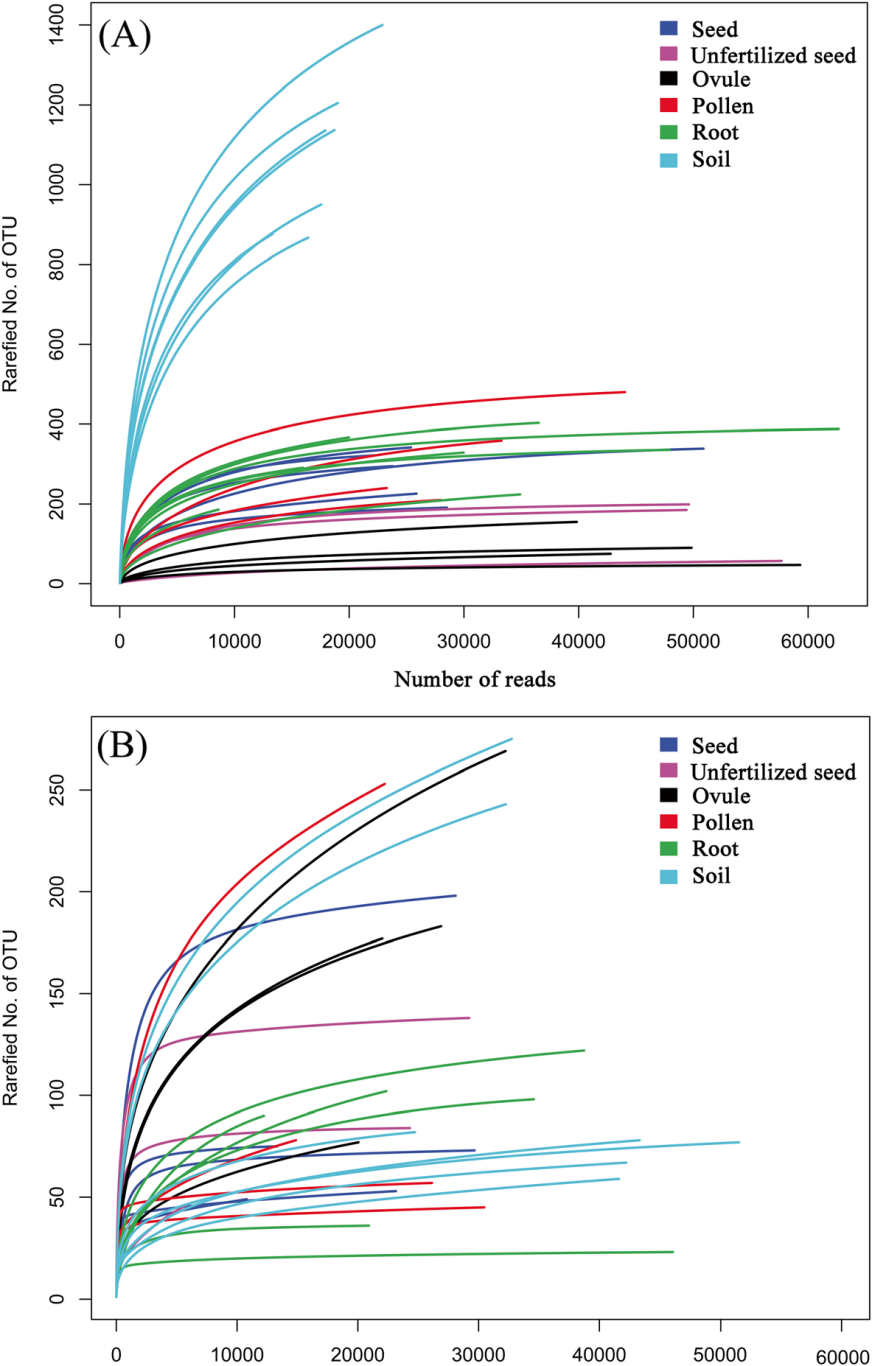
Rarefaction curves of all the 32 samples of *C*. *panzhihuaensis* revealed by 16S rRNA (**a**) and ITS sequences (**b**)

Rarefaction curves were constructed for each individual sample showing the number of observed OTUs relative to the number of total identified microorganism sequences at a 97% sequence similarity cut-off (Fig. 1). For bacterial, microorganisms in soil samples had a higher diversity than the other five sample types (Fig. 1a). In addition, the soil samples exhibited a higher variation in the shape of rarefaction curves as compared to the seeds, pollens, ovules, and unfertilized seeds. The rarefaction curves evaluating the OTU richness per sample generally approached saturation, indicating that the data volume of sequenced reads were reasonable. The soil samples saturated around 700–1070 OTUs and then began to level off, indicating that a greater depth could have revealed more OTUs. Seeds, pollens, and roots showed saturation at about 130–300 OTUs, with ovules and unfertilized seeds below 100 OTUs.

For fungi, only 107 reads were acquired after mapping to Genebank for one unfertilized seed, 1020 and 2483 reads for two root samples from KM, as compared to other samples (more than 6081 OTUs) (Table 1). To reduce the sequencing error, these three samples were excluded in rarefaction curve analysis. Contrary to bacterial results, the fungal rarefaction curves failed to be assigned by sample characters (Fig. 1b). They also got lower saturation values (< 170) than the bacteria. Most of the samples reached the plateau, indicating the sufficiency of the sequencing volume. However, increasing sequencing depth could have retrieved more OTUs for certain samples.

### Alpha diversity

**Fig. 2:**
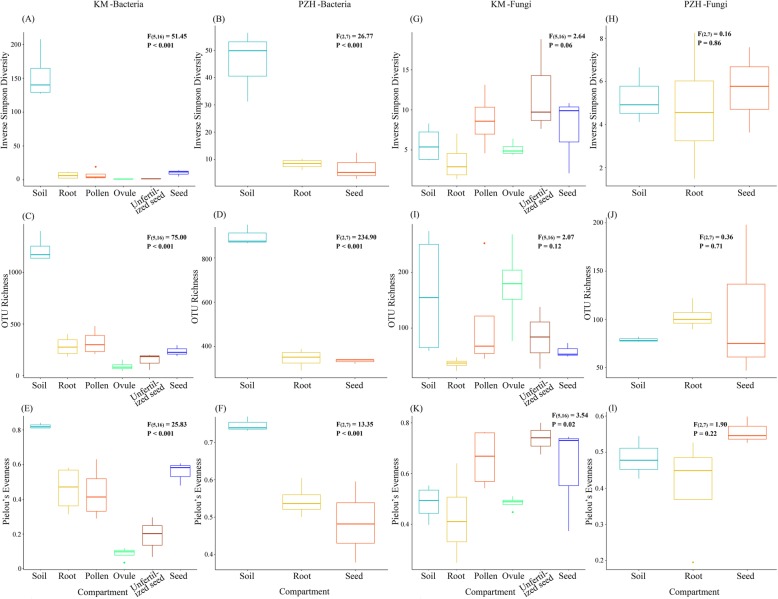
The estimates of alpha diversity indices of the cycad-associated microbial communities from cultural (**a**, **c**, **e**, **g**, **i**, **k**) and natural habitats (**b**, **d**, **f**, **h**, **j**, **l**). **a**–**f** Estimated alpha diversity for bacteria. **g**–**i** Estimated alpha diversity for fungi. **a**, **b**, **g**, **h** The Inverse Simpson diversity index. **c**, **d**, **i**, **j** OTU richness. **e**, **f**, **k**, **l** The Pielou’s evenness. Box plots showed the range of estimated values between 25% and 75%, the median, the minimum and the maximum observed values within each dataset. The overall plant compartment effects (*F*_(DFn, DFd)_ and *p* value) were displayed at the top of each graph

The microbial alpha diversity within each sample was analyzed based on the inverse Simpson diversity index, the OTU richness, and the Pielou’s evenness (Fig. 2). The results of the Levene’s test indicated the homogeneity of variance in the different treatment groups (*p* > 0.05), and the majority results of the Shapiro-Wilk test on the ANOVA residuals (*W* > 0.93, *p* > 0.17) found no violation of normality, except for the Shapiro-Wilk test of the inverse Simpson diversity for KM (*W* = 0.70, *p* = 1.86 × 10^−5^). The Tukey multiple comparisons of means of different compartments at the 95% family-wise confidence level indicated that there was a significant diversity difference between soils and the other five plant organs (adjusted *p* < 0.001). The variations among pollens, ovules, unfertilized seeds, seeds and roots were non-significant with the adjusted *p* values approaching 1.

For the inverse Simpson diversity index estimated based on the 16S rRNA bacterial dataset, the highest bacterial diversity value was observed in soil samples (154 in KM and 45.8 in PZH samples) and consistently decreased diversity estimates in the other five sample compartments (lower than 10.2) (Fig. 2a, b). Similar results were retrieved from OTU richness estimates, with higher richness values detected in soil samples (1220 in KM and 898 in PZH samples) and lower values in all the other five compartments (lower than 350) (Fig. 2c, d). Ovules and pollens exhibited a significant variation of OTU richness (adjusted *p* value < 0.05). For the Pielou’s evenness indices, higher evenness was observed in soil samples (0.822 in KM and 0.746 in PZH samples) as compared to the samples of plant compartments, and the lowest was observed in ovules (0.087) (Fig. 2e, f). Except the variations between soils and the five plant compartments, significant differences were also detected in pairwise comparisons between ovules and roots, unfertilized seeds and roots, ovules and pollens, seeds and ovules, as well as between seeds and unfertilized seeds which were all sampled from the KM habitat (adjusted *p* value < 0.05).

However, for the alpha diversity revealed by ITS sequences, no significant variation of fungal species diversity was observed between groups (Fig. 2g–l).

### Microbial community composition at different taxonomic ranks

**Fig. 3:**
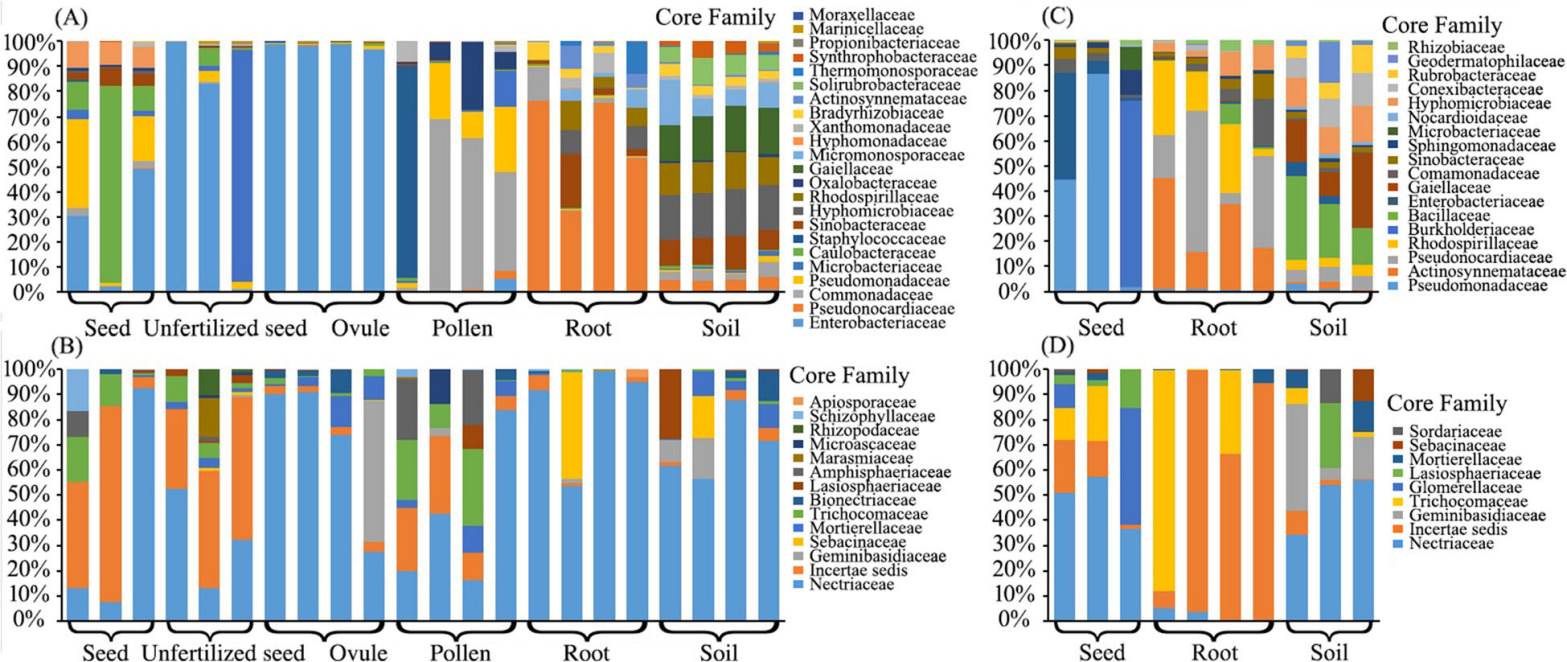
The distributions of core microbial families at different compartments of *C*. *panzhihuaensis*. **a** Core bacterial families derived from KM site. **b** Core bacterial families derived from PZH site. **c** Core fungal families derived from KM site. **d** Core fungal families derived from PZH site

To have a further view of the exact composition of microbiota in different sample types, OTUs were assigned to different taxonomic levels by referring to online datasets. At the Phylum level, all the bacterial sequences could be assigned (Additional file 1A) and 23 phyla were identified. Proteobacteria (61.66%) and Actinobacteria (32.26%) were the most abundant phyla among the 32 samples. Firmicutes (4.21%), Gemmatimonadetes (0.48%), Acidobacteria (0.46%), and Bacteroidetes (0.28%) were identified with low abundance. However, only 77.46% of fungal sequences were assigned to phylum level (Additional file 1B) with Ascomycota dominant (65.29%). The unidentified phylum contributed to 22.54% of sequences. At the Class level, 99.99% of bacterial sequenced were identified, resulting in 68 classes. Gammaproteobacteria (32.20%), Actinobacteria (23.93%), Alphaproteobacteria (15.57%), and Betaproteobacteria (12.40%) were the most dominant classes. For ITS sequences, 72.32% were assigned with 20 fungal classes identified. Among these fungal classes, Sordariomycetes was the most predominant, taking up 49.45% of sequences. At the Order level, 107 bacterial orders and 59 fungal orders were identified, accounting for 98.81% and 71.73% of sequences, respectively. Actinomycetales (23.76%), Enterobacteriales (21.67%), and Burkholderiales (10.92%) were the top three orders in bacterial assemblage. In the fungal order, Hypocreales (43.58%) was the most abundant, followed by Pleosporales (5.95%). At the Family level, 169 (representing 90.25% sequences) bacterial families and 105 (representing 55.92% sequences) fungal families were retrieved. Enterobacteriaceae and Pseudonocardiaceae were the most predominant bacterial families (Fig. 3a, b) and Nectriaceae was the dominant fungal family (Fig. 3c, d). At the lower taxonomic ranks, only 49.25% of bacterial sequences and 31.88% of fungal sequences were assigned to the generic level. Consequently, the beta diversity index was evaluated at two phylogenetic levels, the Family level and the OTU level.

### Beta diversity

**Fig. 4:**
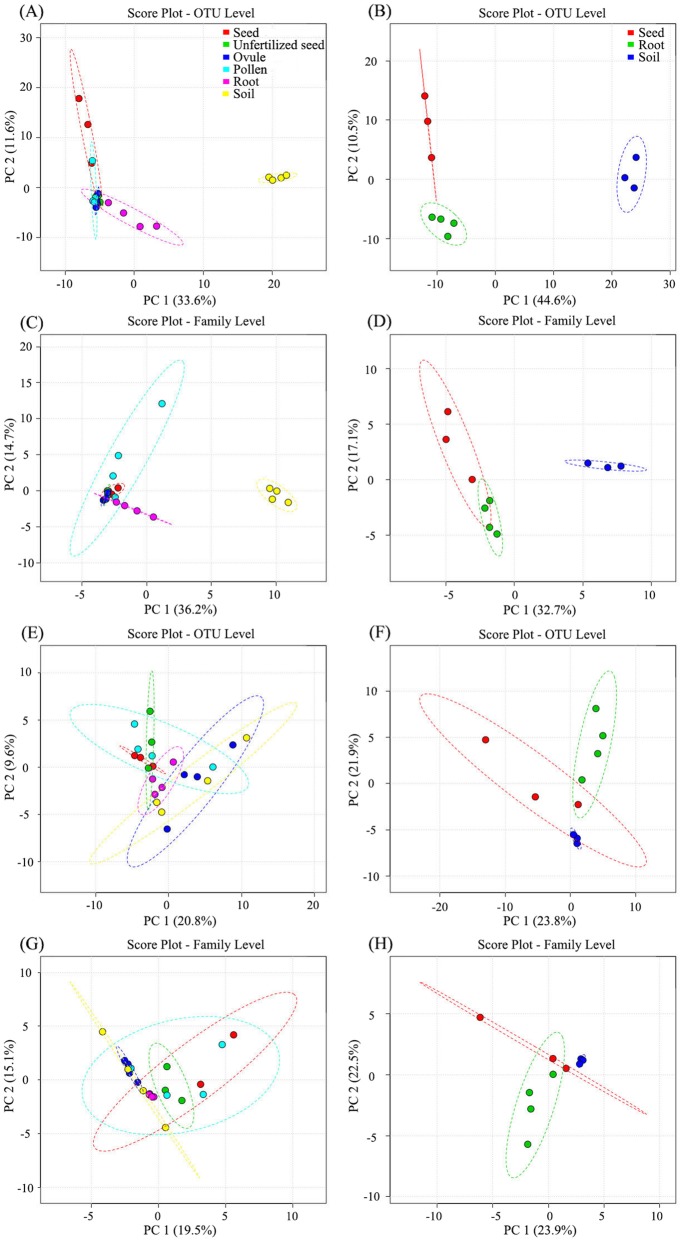
Principle component analysis of microbial community composition among different sample types of *Cycas panzhihuaensis* at OTU (**a**, **b**, **e**, **f**) and Family (**c**, **d**, **g**, **h**) level. **a**–**d** Bacterial community composition from KM (**a**, **c**) and PZH (**b**, **d**) site. **e**–**h** Fungal community composition from KM (**e**, **g**) and PZH (**f**, **h**) site. **a**, **c**, **e**, **g** Shared the same legend, and the same to (**b**, **d**, **f**, **h**). The ellipsis represented 95% confidence intervals

To compare the composition of identified community members within different compartments and identify main factors driving community composition, principal component analyses (PCA), hierarchical cluster analyses (HCA), as well as heatmap analyses of microbial community structure were performed based on the Bray-Curtis dissimilarity matrix on the website METAGENAssist with the ‘Pearson’ distance measure and the ‘ward’ clustering algorithm. Whether at the all ranks level (OTU), or at the family level, strong clustering of bacterial communities was uncovered between soils and the plant compartments by principal component analysis (Fig. 4a–d), but this was not the case of the fungal community (Fig. 4e–h). At the OTU level, PC 1 explained 33.6% of the total variation among samples collected from KM, and PC 2 explained 11.6% (Fig. 4a). Soils exhibited a significant difference of community composition comparing with the other five plant compartments. Seeds and roots showed a certain degree of community structure variation with ovules, pollens, and unfertilized seeds. For PZH individuals, PC 1 explained 44.6% and PC 2 explained 10.5% of the total bacterial variations (Fig. 4b). Similar to the results of KM samples, a significant variation of community composition between soils and plant compartments and between seeds and roots, was also detected. At the family level, 36.2% variations among KM samples were explained by PC 1 and 14.7% by PC 2 (Fig. 4c). Soils showed significant variation of bacterial community composition with plant samples. Among plant samples, pollens and roots displayed some degree of community structure variations. For PZH samples, PC 1 explained 32.7% and PC 2 explained 17.1% of the total variations (Fig. 4d). Soil exhibited the variation of community structure with seeds and roots. The variation between seeds and roots was non-significant.

**Fig. 5:**
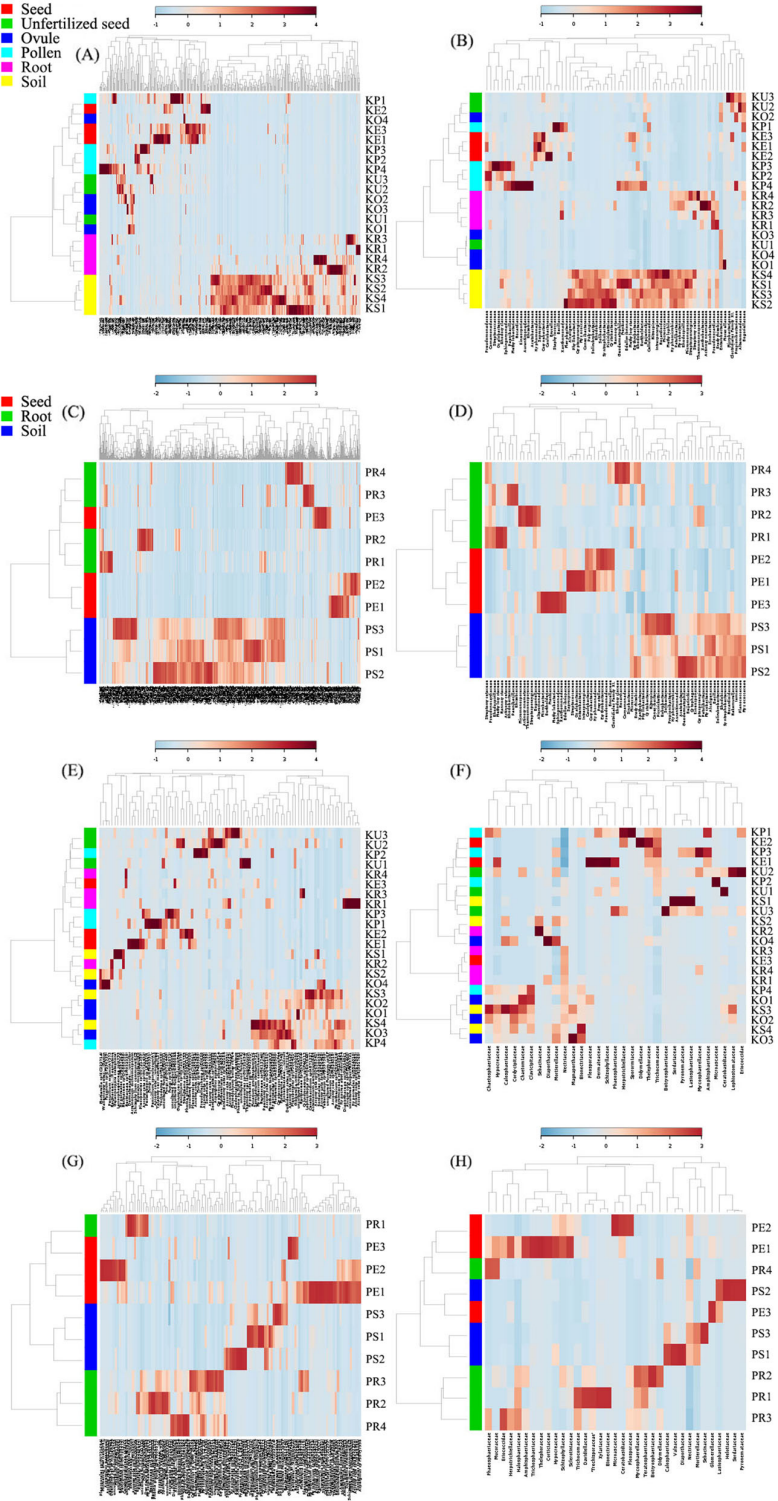
The results of hierarchical cluster analyses and heatmap analyses among different sample types of *C*. *panzhihuaensis* at the OTU (**a**, **c**, **e**, **g**) and Family (**b**, **d**, **f**, **h**) level. **a**–**d** HCA (left) and heatmap (right) results based on 16S rRNA sequence derived from KM (**a**, **b**) and PZH (**c**, **d**) habitats. **e**–**h** Results derived from KM (**e**, **f**) and PZH (**g**, **h**) habitats based on ITS sequences. **a**, **b**, **e, f** shared the same legend, and the same to (**c**, **d**, **g**, **h**).

The results of HCA and heatmap based on the 16S rRNA dataset support the above analyses (Fig. 5a–d). Two clusters were clearly revealed, one grouped by soil samples and the other contained individuals from plant compartments. Within the plant compartment cluster, the grouping was ambiguous and not exactly matching with their respective plant compartments. For KM, root samples clustered together and separated with the above-ground plant compartments (Fig. 5a). Samples of ovules, pollens, unfertilized seeds, and seeds clustered together and could not be distinguished individually at the OTU level. However, at the family level, root samples were embedded within the above-ground compartments (Fig. 5b). For PZH, complete clustering was revealed according to the sample types at the family level (Fig. 5d), but not at the OTU level (Fig. 5c). Soil samples were clearly distinguished from seeds and root samples. However, no such clear clustering structure of fungal communities was observed, although roots from PZH exhibited a certain degree of microbial community variation with soils and seeds (Fig. 5e–h).

Comparable results were generated by pairwise Adonis (Table 3). For KM samples, a nearly similar result was retrieved at the OTU and Family level. Soils and roots respectively exhibited significant dissimilarity of bacterial community composition comparing with the other four sample types (0.37 < *R*^2^ < 0.97, *p* < 0.05). When comparing the bacterial community between ovules and pollens, significant variation was also detected (*R*^2^ = 0.40 at the OTU level, *R*^2^ = 0.66 at the Family level; *p* < 0.05). In addition, seeds and ovules showed bacterial microbiota dissimilar from each other at the Family level (*R*^2^ = 0.69, *p* < 0.05), while for fungal community, most sample types showed similar microbial community composition with *R*^2^ < 0.30, *p* > 0.05 at the OTU level and *R*^2^ < 0.36, *p* > 0.05 at the Family level. For samples collected from the PZH site, whether at the OTU level or at the Family level, a significant variation of bacterial and fungal microbial similarity was observed between roots and soils (0.46 < *R*^2^ < 0.58, *p* < 0.05). The differentiation between seed- and root-associated microbiomes was also remarkable with fungal at the Family level as a except (*p* = 0.06).

**Table 3:** PERMANOVA and pairwise comparisons of microbial community variations among different plant compartments of *C*. *panzhihuaensis* at two phylogeny levels

	Phylogenetic level pairwise.adonis output	16S				ITS			
		OTU level		Family level		OTU level		Family level	
		*R*^2^	*p*	*R*^2^	*p*	*R*^2^	*p*	*R*^2^	*p*
KM	Seed vs. Unfertilized seed	0.3814	0.100	0.3073	0.200	0.2295	0.200	0.1370	0.800
	Seed vs. Ovule	0.4365	0.065	0.6859	0.023*	0.3716	0.032*	0.3213	0.124
	Seed vs. Pollen	0.4087	0.055	0.3794	0.062	0.2397	0.054	0.0659	0.916
	Seed vs. Root	0.4670	0.028*	0.6427	0.029*	0.1991	0.327	0.3609	0.096
	Seed vs. Soil	0.7039	0.028*	0.7335	0.029*	0.2973	0.032*	0.3458	0.092
	Unfertilized seed vs. Ovule	0.0994	0.740	0.2571	0.137	0.2984	0.057	0.5078	0.029*
	Unfertilized seed vs. Pollen	0.3580	0.054	0.3754	0.089	0.1750	0.346	0.2039	0.316
	Unfertilized seed vs. Root	0.4027	0.025*	0.5622	0.029*	0.1954	0.235	0.5728	0.038*
	Unfertilized seed vs. Soil	0.6189	0.024*	0.6509	0.030*	0.2475	0.082	0.5067	0.030*
	Ovule vs. Pollen	0.3994	0.031*	0.6626	0.023*	0.2312	0.143	0.2569	0.084
	Ovule vs. Root	0.4324	0.034*	0.8565	0.024*	0.2017	0.112	0.1439	0.438
	Ovule vs. Soil	0.6302	0.028*	0.9761	0.023*	0.0912	0.768	0.0440	0.967
	Pollen vs. Root	0.3712	0.021*	0.5319	0.028*	0.2127	0.057	0.3208	0.095
	Pollen vs. Soil	0.5655	0.024*	0.6045	0.021*	0.2107	0.176	0.2703	0.113
	Root vs. Soil	0.5458	0.029*	0.7277	0.029*	0.1576	0.248	0.1292	0.585
PZH	Seed vs. Root	0.3299	0.023*	0.4662	0.035*	0.3706	0.032*	0.4463	0.060
	Seed vs. Soil	0.5427	0.100	0.5631	0.100	0.3566	0.100	0.4011	0.100
	Root vs. Soil	0.4614	0.021*	0.5833	0.023*	0.5433	0.031*	0.5104	0.029*

*R*^2^: adonis test statistic. Significance level: *p < 0.05

### Top members of the microbiome among compartments

Twenty-three core bacterial families (Fig. 3a) and 14 fungal families (Fig. 3c) for KM and 18 core bacterial (Fig. 3b) and nine fungal families (Fig. 3d) for PZH were identified in this study (Additional file 2). Most of the families displayed a significant compartment effect with p values < 0.05 (Additional file 2). For seed samples, Caulobacteraceae was the most abundant bacterial family (relative abundance = 31.20%) for KM and Burkholderiaceae (32.34%) for PZH. However, these two families were not significantly differed among sample types (*p* > 0.05). A significant enrichment of Enterobacteriaceae (23.09%) and Pseudomonadaceae (15.68%) were observed in seeds from KM. Pseudomonadaceae was also remarkably enriched in seed samples (26.15%), as compared to the root and soil samples from PZH. For the unfertilized seeds, Enterobacteriaceae (61.41%) was significantly abundant (*p* < 0.05), followed by the enrichment of Microbacteriaceae (28.21%). An extremely high relative abundance of sequences from Enterobacteriaceae (96.75%) was observed in ovule samples, whereas other families contained far fewer reads. Differing from the abovementioned results, Comamonadaceae (33.24%) and Staphylococcaceae (20.25%) were dominant in pollen samples with a significant variation of Comamonadaceae between pollen and the other five sample types (*p* < 0.05). Pseudonocardiaceae remarkably enriched (*p* < 0.05) in root samples from KM with a relative abundance of 51.10%. However, for root samples from PZH, Actinosynnemataceae (21.86%) was the dominant family followed by Pseudonocardiaceae (15.70%) and Rhodospirillaceae (14.44%). A significant variation of Rhodospirillaceae was observed among seeds, roots, and soil samples from PZH (*p* < 0.05). For the soil samples, most of the sequences were unidentified, 28.77% for KM and 32.05% for PZH. Gaiellaceae was the only family shared by KM and PZH samples with a minor extend relative abundance detected in soil samples (7.91% for KM and 10% for PZH).

For the core fungal microbiomes, most of the sequences were unidentified among the six sample types (Additional file 2). Nectriaceae was extremely abundant in seeds (34.31%), ovules (45.06%), pollens (23.40%), roots (83.40%), and soils (41.25%) from KM. For unfertilized seeds, *Incertae sedis* was remarkably enriched (*p* < 0.05) with high relative abundance (23.16%) as compared to the other compartments. Trichocomaceae was another fungal family that displayed significant variation of relative abundance among the sample types (*p* < 0.05). However, the relative abundance of this family was very low, with the highest value observed in pollens (4.11%). For samples from PZH, a significant enrichment of Nectriaceae (*p* < 0.05) was observed in seeds (13.42%) and soils (33.89%), as compared to roots (0.31%). *Incertae sedis* was abundant in root samples (19.72%).

### Indicator species analysis

When considering the number of indicator species, a total of 19 indicator bacterial families were identified. Fifteen were found in seeds, 13 in unfertilized seeds, ten in ovules, 15 in pollens, 16 in roots, and 17 in soil samples from the KM site (Table 4). Pseudonocardiaceae, Hyphomicrobiaceae, Rhodospirillaceae, Comamonadaceae Xanthomonadaceae, Sinobacteraceae, and Enterobacteriaceae were common in KM individuals. For samples from the PZH site, each sample type contained 16 indicator bacterial families but not a single member of Chitinophagaceae, Halothiobacillaceae and Moraxellaceae which presenting at the KM site, was detected. The number of indicator species revealed by ITS marker was a little higher than the 16S rRNA sequences (25 in total). However, for each compartment, it was a little lower: six in seeds, nine in unfertilized seeds, 16 in ovules, 14 in pollens, eight in roots and 17 in soil samples from KM site, and nine in seeds, seven in roots, and five in soils from PZH site (Table 4). Nectriaceae and Hypocreaceae were the only two fungal families presenting in all 32 sampled individuals.

**Table 4:** Results of indicator species analysis for samples from different habitats

	Indicator species	KM						PZH			Indicator value	*P* value
		Seed	Unfertilized seed	Ovule	Pollen	Root	Soil	Seed	Root	Soil		
16S	Nocardioidaceae	0.05%	0.01%	0	0.72%	0.24%	4.71%	0.20%	0.14%	0.65%	0.945	0.002**
	Pseudonocardiaceae	0.11%	0.01%	0.01%	0.60%	53.85%	2.17%	0.04%	18.88%	2.78%	0.856	0.001***
	Hyphomicrobiaceae	0.57%	0.08%	0.01%	0.19%	4.32%	8.37%	0.31%	4.84%	6.33%	0.786	0.007**
	Rhodospirillaceae	0.10%	0.03%	0.01%	0.01%	5.01%	5.95%	0.06%	14.18%	2.16%	0.748	0.019*
	Comamonadaceae	1.90%	0.35%	0.16%	36.56%	3.92%	1.87%	2.54%	4.83%	0.44%	0.862	0.002**
	Xanthomonadaceae	0.78%	0.12%	0.01%	2.32%	2.65%	0.73%	2.65%	0.44%	0.18%	0.817	0.004**
	Rhizobiaceae	0.10%	0	0.01%	0.40%	0.06%	0.18%	0.64%	1.46%	0.21%	0.749	0.016*
	Bradyrhizobiaceae	0.04%	0.02%	0	0.06%	3.21%	1.71%	0.37%	0.97%	1.38%	0.612	0.049*
	Patulibacteraceae	0.03%	0	0	0.16%	0.82%	1.55%	0.04%	0.99%	1.40%	0.791	0.002**
	Mycobacteriaceae	0	0	0	0.03%	0.61%	0.69%	0.73%	0.99%	1.92%	0.791	0.002**
	Bacillaceae	0.20%	0.04%	0	0.78%	0.01%	0.11%	0.13%	1.95%	12.17%	0.785	0.002**
	Sinobacteraceae	4.47%	0.25%	0.09%	0.10%	6.01%	4.95%	1.78%	3.34%	1.18%	0.778	0.001***
	Phyllobacteriaceae	0.09%	0	0.01%	0	0.18%	0.38%	0.18%	0.33%	0.31%	0.759	0.019*
	Micromonosporaceae	0	0	0	0.01%	2.98%	4.77%	0.15%	4.96%	1.63%	0.755	0.006**
	Chitinophagaceae	0	0	0	0	0	0	0	0	0	0.752	0.021*
	Microbacteriaceae	1.65%	29.71%	0	2.71%	0.02%	0.46%	2.67%	0.02%	0.09%	0.744	0.004**
	Halothiobacillaceae	0	0.01%	0	0	0	0	0	0	0	0.707	0.006**
	Enterobacteriaceae	24.40%	59.34%	96.66%	1.19%	0.03%	0.12%	11.92%	0.26%	1.59%	0.679	0.018*
	Moraxellaceae	0	0.01%	0.19%	0	0	0	0	0	0	0.672	0.009**
ITS	Diaporthaceae	0	0	2.59%	0.11%	1.25%	0.39%	0.04%	0.03%	3.29%	0.945	0.002**
	Geminibasidiaceae	0	0.07%	10.48%	0.47%	0.38%	3.91%	0	0	15.98%	0.856	0.001***
	Sebacinaceae	0	0.37%	0.03%	0	9.14%	3.37%	0.13%	0	3.39%	0.786	0.007**
	Togniniaceae	0	0	0.01%	0	0	0.02%	0	0	0	0.748	0.019*
	Incertae sedis	0	0	0	0	0	0	0	0	0	0.862	0.002**
	Trichocomaceae	3.07%	2.58%	1.08%	4.02%	0.11%	0.61%	2.71%	4.66%	2.17%	0.817	0.004**
	Sclerotiniaceae	0	0.07%	0	0.29%	0	0	0.26%	0.03%	0	0.749	0.016*
	Cystobasidiaceae	0	0.04%	0	0	0	0	1.21%	0.02%	0	0.612	0.049*
	Fomitopsidaceae	0.08%	0	0	0	0	0	0	0	0	0.791	0.002**
	Coniochaetaceae	0	0	0	0.01%	0	0.01%	0	0	0	0.791	0.002**
	Magnaporthaceae	0	0	0.64%	0.04%	0	0.19%	0	0	0	0.785	0.002**
	Nectriaceae	25.29%	12.25%	43.61%	22.58%	61.22%	42.56%	14.34%	0.35%	32.04%	0.778	0.001***
	Bionectriaceae	0.22%	0	2.41%	0.68%	0.07%	2.65%	0	0.06%	0	0.759	0.019*
	Vibrisseaceae	0	0	0.12%	0	0	0.16%	0	0	0	0.755	0.006**
	Hypocreaceae	0.47%	0.21%	0.72%	0.55%	0.10%	0.88%	0.12%	0	0	0.752	0.021*
	Chaetosphaeriaceae	0	0.02%	0.22%	0.27%	0	0.33%	0	0	0	0.744	0.004**
	Pluteaceae	0	0	0	0	0	0	0	0	0	0.707	0.006**
	Clavicipitaceae	0	0	0.05%	0.04%	0	0.05%	0	0	0	0.679	0.018*
	Myxotrichaceae	0	0	0	0	0	0	0.01%	0	0	0.672	0.009**
	Chaetomiaceae	0	0.04%	0.18%	0.05%	0	0.14%	0.03%	0	0	0.652	0.032*
	Tubeufiaceae	0	0	0.02%	0.01%	0	0.01%	0	0	0	0.631	0.024*
	Ustilaginaceae	0	0	0.01%	0	0	0	0	0	0	0.612	0.028*
	Apiosporaceae	0	0	0.02%	0	0.66%	0.04%	0	0	0	0.61	0.025*
	Mucoraceae	0	0	0	0.01%	0	0	0	0.01%	0	0.605	0.029*
	Halosphaeriaceae	0	0	0	0	0	0.01%	0	0	0	0.601	0.018*

Significance levels: *p ≤ 0.05; **p ≤ 0.01; ***p ≤ 0.001

When considering the predominant indicator species among sample types, no matter where the samples were from, seeds were primarily dominant by Enterobacteriaceae (indicator value = 0.679, *p* = 0.018) and roots by Pseudonocardiaceae (indicator value = 0.856, *p* = 0.001). Bulk soils from KM mainly contained Hyphomicrobiaceae (indicator value = 0.786, *p* = 0.007), whereas the dominant member of soils from PZH was Bacillaceae (indicator value = 0.785, *p* = 0.002). For the fungal community, the key indicator species for seeds, roots, and soils was Nectriaceae (indicator value = 0.778, *p* = 0.001) except roots from PZH which was dominant by Trichocomaceae but with low relative abundance (4.66%) (Table 4).

### Venn figure comparison

**Fig. 6:**
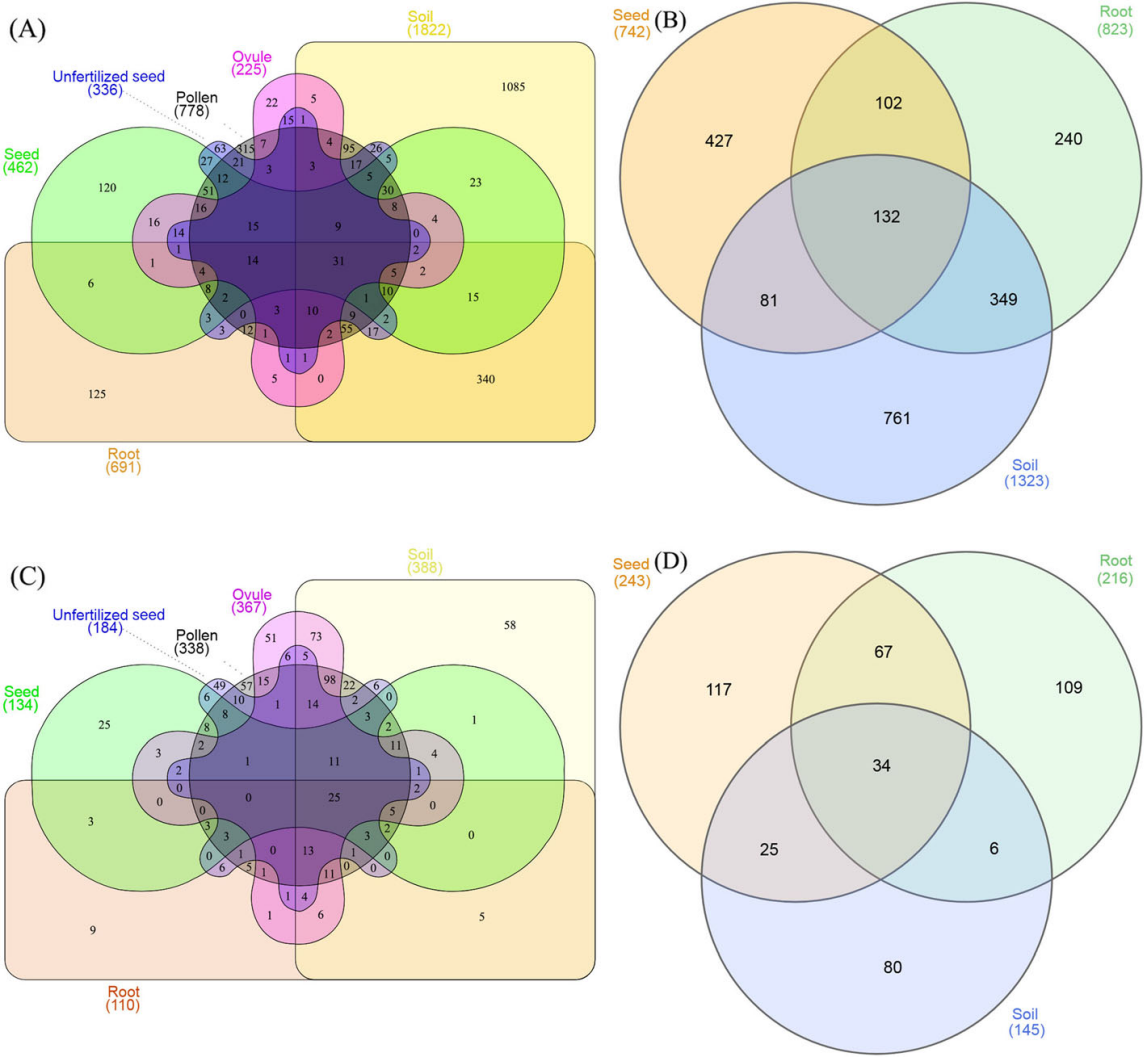
Venn diagrams demonstrated the overlaps of cycad-associated microbiome among different compartments of *C*. *panzhihuaensis* at the OTU level. **a**, **b** Bacterial OTUs from KM (**a**) and PZH (**b**) habitats. **c**, **d** Fungal OTUs from KM (**c**) and PZH (**d**) habitats

To provide a complete overview of the OTU distribution within different compartments, the number of OTUs uniquely identified in each sample type as well as the OTUs shared by different compartments, were calculated. A total of 2728 bacterial OTUs were generated in KM samples with 1.14% shared among the six sample groups (Fig. 6a). Approximatively 0.51% of the total OTUs were commonly present in the five plant organs, and 39.77% were exclusively found in soil samples compared to pollens (11.55%), roots (4.58%), seeds (4.40%), unfertilized seeds (2.31%), and ovule samples (0.81%). A high overlap (12.46%) of OTUs from soils and roots was also observed. For PZH individuals, 6.31% of total bacterial OTUs (2092) were shared among the three sample types (Fig. 6b). Nearly 36.38% of OTUs were particularly occupied by soils, 20.41% by seeds, and 11.47% by root samples.

However, the number of fungal OTUs retrieved from KM samples was 665 with 3.76% shared among different compartments (Fig. 6c). Specifically, 58 private OTUs were separately identified in soils, as compared to pollens (57), ovules (51), unfertilized seeds (49), seeds (25), and roots (9), while a total of 438 fungal OTUs were detected in PZH samples (Fig. 6d). About 7.76% of the OTUs were shared among seeds, soils, and root samples. Most of the OTUs were privately occupied with 117 in seeds, 109 in roots, and 80 in soils.

## Discussion

### Each compartment represents a unique ecological niche for microbiome of *Cycas panzhihuaensis*

As revealed by rarefaction curves and the alpha diversity index, a significant differentiation of species diversity between bulk soils and plant organs of *C*. *panzhihuaensis* was uncovered, which is in concordance with the general views of microbial colonization [13]. Soil serves as one of the richest microbial ecosystems on Earth, providing ideal habitats for various microbial lineages [9]. The estimated bacterial diversity within 0.5 g of soil was higher than 2000 species [32–35]. However, species diversity inside plant compartments (endophytes) or attached to the surface of samples (epiphytes) was much lower, mainly owing to the following factors. On the one hand, the process of microbial colonization and formation of stable communities in plant tissues was highly variable and more complex than expected, constrained by numerous biotic and abiotic factors, such as the host plant’s innate immune system and their response to microbial colonization. On the other hand, even if successful invasion happened, the ability to accommodate the microenvironments in plant tissues, including the limited intercellular space, the unevenly allocated nutrients coupled with temperature and humidity heterogeneity, should be finely developed. The great loss of species diversity from soil to plant tissues (especially ovules and unfertilized seeds) indicated that only a limited number of bacterial microbes could keep a symbiotic lifestyle with a host plant (loss of diversity and richness) and finally become dominant endophytic assemblages (loss of evenness).

Whether at the OTU level, or at the family level, remarkably variation of bacterial community structure was disclosed between soil and plant compartments by PCA, HCA, and heatmap analyses. Nevertheless, the differentiation of community structure among plant compartments was non-significant and there was no sign of fungal community variation among sample types. As mentioned previously, microbiota in soil may be supplied as the microbial pool and root microbiomes are largely renewed from soil microbes by horizontal transmission. For the above-ground plant tissues, two types of microbial sources may occur, namely horizontal transmission from atmosphere and vertical transmission via seeds. The differentiation of niche, especially between soil and plant tissues, gave rise to the variation of community structures of bacterial microbiomes. However, for fungal community, no such variation was detected between soil and plant compartments. Similar results have been reported in model species poplar [13] and *Arabidopsis thaliana* [36, 37], as well as non-model species *Agave* [7, 38], cacti [39], and other plant species [40–42]. Phylogenetic profiling uncovered that the key factor driving fungal microbiome in agave plants is the biogeography of the host, with a contrast that bacterial microbiome is primarily reshaped by plant tissues [38, 43]. Each of the plant microenvironments or ecological niches provides relevant biotic and abiotic gradients such as availability of soluble organic compounds and oxygen. The niche-specific settings among different plant tissues were much more similar than in comparison with rhizosphere soils, resulting in assembled bacterial taxa [13]. However, it is likely that habitat-specific features (i.e., climate and soil types) drive assemblages of distinct plant-associated fungal communities with a certain degree of functional redundancies across sites [38].

### Slight differentiation of microbiomes was observed concerning the habitat variation (KM vs. PZH)

According to the indicator species analysis, there was almost no differentiation of dominant microbiomes with regard to the geography of the host cycad, but host/niche-specific bacterial taxa were identified. It has been well established that an array of environmental and host-associated factors jointly influence host plant microbiomes, such as climate, soil type, plant genotype, and its biogeography. However, factors driving bacterial and fungal communities differ to certain extents. The study of Agave plants demonstrated that the associated bacterial communities were chiefly influenced by plant compartments or sample types, whereas the rhizosphere soil, the root, and leaf endosphere were clearly distinct from one another. However, the fungal communities were varied by geographic distance of the host [43].

In this study, the climate conditions in PZH (natural habitat) were characterized by dry and hot weather with an annual average temperature of 21 °C and an annual precipitation of 800 mm [44]. The weather characters in KM (cultural habitat) were much more pleasant, featuring warm and wet conditions with an annual average temperature about 15.09 °C, and an annual precipitation around 994.69 mm [45]. The variation of climate conditions and possibly soil properties, coupled with cultivation practices, gave rise to the differentiation of local microbiomes, leading to the formation of distinct and highly diverse soil bacterial microbiota. However, limited by its dispersal capability, fungal endemism may be a community-shaping force working at multiple scales and in multiple habitats [43]. Interestingly, the variation of root fungal communities was detected between KM and PZH sites, although with low relative abundance (Trichocomaceae, 4.66%). Both Nectriaceae and Trichocomaceae belong to the Hypocreales order in the Sordariomycetes class of the phylum Ascomycota. Species from these two families are common plant endophytes [46–48] and have been reported to confer plant fitness benefits [49–51]. It is likely that the host cycad selected functional groups rather than taxonomic groups of fungal microbiomes [1].

As a conclusion, we deem that the slight differentiation of *C*. *panzhihuaensis* microbiomes between its natural and managed habitats may reflect a certain degree of microbial conservation regulated by host selection [52].

### Niche preference exists for core bacterial microbiome of *Cycas panzhihuaensis*

Structural variability and niche differentiation in the rhizosphere and endosphere bacterial microbiome of field-grown poplar trees have been revealed by Becker et al. with the conclusion that each niche inside plant organs as well as bulk soils represent unique habitat for the bacterial communities [13]. The plant host-specific traits, including the internal organizational structures, physical and chemical characteristics, specific metabolic pathways, and genetic products [53, 54], boost the differentiation of bacterial lineages corresponding to the niche types. According to the core bacterial microbiome analysis in present research, seed was primarily dominated by Caulobacteraceae and Enterobacteriaceae. Enterobacteriaceae was also the key family for both unfertilized seeds and ovules. The dominant member of the pollen samples was Comamonadaceae. The core bacterial microbiome of roots was dominated by Pseudonocardiaceae, and Hyphomicrobiaceae for soil samples. The similarity of bacterial communities among seeds, unfertilized seeds, and ovule samples can be explained by the fact that these three plant organs are maternally originated and share some genetic materials. The core bacterial communities, especially the dominant taxa, shared by these three sample types were highly likely to be vertically transmitted and displayed species conservation to a certain degree. For the dominant strains exclusively obtained by each compartment, the host specificity or niche differentiation may exert a more powerful effect.

However, for the core fungal community, five of the six sample types were dominated by species from family Nectriaceae (with unfertilized seeds as an exception which was dominated by *Insertae sedis*), suggesting that the differentiation of plant compartments did not exert impact on fungal communities. The resemblance of core fungal communities among sample types verified the previous finding that plant compartments did not play a significant role on fungal assemblages [38, 43], which referenced the evolutionary conservation of plant-associated fungal microbiomes.

### The origin and transmission mode of microbiome in *Cycas panzhihuaensis*

Soil environment hosts a plethora of microorganisms and has been widely acknowledged as the biodiversity and hotspot for studying the origin of plant-associated microbiomes [7]. There are two different ways concerning the origin and transmission mode of microbiomes in plants: namely the horizontal and vertical transmission pathways. Most plant-associated microbiomes were horizontally transmitted from soil-borne microorganisms. First, rhizodeposition and root exudation produced by the host plant in the rhizosphere motivated chemoattraction, inducing the soil and/or rhizosphere microbiomes to break through root surface barriers [55] and inhabit the inside root tissues. After entering the root, the microbes are translocated systemically from the underground parts of the plant to the aboveground compartments, and finally reach the reproductive organs and seeds. Two main pathways have been proposed for microbiome spreading throughout the plant tissues. One is through the root xylem vessels of the host plant with the assistance of flagella and the plant transpiration stream. The other pathway uses the nutrient-rich intercellular spaces by secreting cell wall degrading enzymes [56, 57]. Seed microbiomes, however, are not exclusively soil derived. Caulosphere, phyllosphere, anthosphere, and carposphere are all considered to be the alternative sources of seed/plant microbiomes. Microbiomes inside plant tissues can be transferred from the maternal plant through the funiculus and chalaza into the seed endosperm as well as via the micropyle [56]. Furthermore, microbial associations with gametes (i.e., pollens) have been reported in pine [58, 59], and may contribute to the colonization of the embryo and endosperm as a result of pollination of the ovule [60]. Generally, the traditional route of microbes from soil/air-borne conditions to microenvironments in various plant tissues includes penetration through the epidermis of different tissues and ovary walls, systemic infection via the vascular system, or penetration into the ovule via pollen germination inside the stigma [61].

The vertical transmission process from seed to progeny has been frequently reported in different plant taxa [62, 63], especially the grasses [64, 65], with the transmission efficiency remaining a matter of debate [8]. Microorganisms hosted by seeds contribute to seed germination, seedling establishment, plant growth, and fitness [2, 5, 66]. Two major routes existed for the seed endophytes to infect the next generation: exiting the seed and reentering through plant surface or remaining inside the seed and spreading into different plant tissues with the development of seedlings [67]. However, the vertical transmission process is far from perfect and microbes will be lost at all possible stages during plant growth [64]. In other words, only a small proportion of vertically transmitted microbes will be remained in the next generation.

In the case of *C*. *panzhihuaensis,* both horizontal and vertical transmission pathways may exist. The results of PCA and HCA delineated a significant variation of community composition of soil microbiome as compared to the other five plant compartments. Among plant compartments, niche-specific bacterial communities have been uncovered. Given the species diversity, the highest level of Shannon diversity index was observed in soil samples, followed by seeds, with the lowest level found in ovules and unfertilized seeds. This result was not unexpected and could be explained by the fact that bulk soil has been confirmed to embrace the highest level of bacterial diversity among the Earth’s ecosystems [68]. Although the root was the first agent for soil-borne microbial assemblages to invade the plant tissues, and though a lot of research has ascertained the diversity and abundance of microbial species in roots, a higher level of species diversity was observed in seeds of *C*. *panzhihuanesis* than in roots in the current study. The existence of different microorganism sources added to the total number of bacterial species in seeds. Bacteria within ovules and pollens have previously been detected in 27 plant species by Mundt and Hinkle [69]. Higher bacterial diversity of pollens than ovules may partly result from the non-thoroughness of the sterilization procedure for pollens, due to their small size. In addition, cycads are an allogamous plant. Microbes detected in pollens may also include bacteria from air particles or pollinators [8, 70]. Unfertilized seeds displayed a similar level of species diversity as ovules, which is significantly less than the other four sample types. The protective mechanism of the ovule against exogenous microbials provided by various proteins may account for this phenomenon [71–73].

The large overlap between core bacterial assemblages and indicator species of microbiomes across different compartments demonstrated that endophytic competence and dealing with niche-specific plant settings is reserved for a certain group of bacterial taxa. For the common species identified by the Venn diagram in this study, they may be horizontally derived from soils and kept in each sample type during the transmission process. Alternatively, it is also possible that they are vertically taken over from seeds. However, the exact transmission mode of the identified taxa cannot be decided by this study and further research is needed.

### Potential ecological functions of dominant microbiota in *Cycas panzhihuaensis*

The co-occurrence of certain microbial groups resulting from indicator species and core microbiome analyzes of *C*. *panzhihuaensis* indicates the importance of these microbes. Therefore, the potential ecological roles of the most dominant species are discussed in this section. Among the identified bacterial species, *Xylophilus ampelinus* was the most abundant strain which was only present in pollens and soils. The less extent species, *Variovorax paradoxus*, was found in every compartment of the host *C*. *panzhihuaensis*. *V*. *paradoxus* has been confirmed as a member of plant growth-promoting bacteria in various plants, such as *Anthurium andraeanum* [74], soybean [75] and sugar beet (*Beta vulgaris* L.) [76, 77]. It benefits host plants by enhancing the plant’s disease resistance and stress tolerance [76, 77], aiding in nutrient availability and assimilation [78, 79]. Some strains were capable of accumulating rare-earth elements, reducing their concentrations [80]. The most dominant fungal species was *Haematonectria haematococca*, which was mainly occurred in soils and ovules. *Gibberella intricans* (asexual name was *Fusarium equiseti*) was another strain widely appeared among *C*. *panzhihuaensis* compartments with high relative abundance. These microbes were the naturally occurring endophytes in cereals [81] and other plants [82], and have been found to be non-pathogenic to the host sainfoin [83]. Specifically, endophytic *F*. *equiseti* has been treated as an effective biocontrol agent against root rot disease of tomato [84] and pea [85], stimulating plant growth.

Given the natural characteristic of arid and harsh conditions where *C*. *panzhihuaensis* dwelled, the cycad-associated microbiomes may contribute to the host’s growth and environmental adaptation by nutrition supplement, phytopathogen resistance, and stress tolerance.

## Conclusions

The plant-associated microbiota confers resistance to (a) biotic stress and promotes plant growth and fitness. The cycads represent as an important model to study the associations between plants and microbial communities across tissue-level niches, given their adaption to arid and infertile soil environments. Here, highly diverse and finely structured microbial communities were detected among different sample types of *C*. *panzhihuaensis*. Niche-specific taxa were also observed. However, biogeography did not play a role in the differentiation of microbiomes between natural and cultivated habitats as indicated by previous studies. Further, the origin and transmission mode of microbiomes in *C*. *panzhihuaensis* has been proposed. The dominant microbiome of cycad species may benefit the host by nutrient supply and seedling growth, as well as in drought tolerance. Overall, this study provides a holistic understanding of microbiomes associated with different compartments of a relic gymnosperm plant. Hopefully, these efforts will lend a baseline to further deepen our knowledge of plant-microbe interactions in arid and nutrient-poor conditions.

## Methods

### Sample collection

Two health populations of *C*. *panzhihuaensis* were selected for sampling. One was a cultivated population from Kunming Botanical Garden in Yunnan, China (KM), where pollens, ovules, seeds, unfertilized seeds, coralloid roots, and the attached rhizosphere soils were collected from July to September 2016. The other was a natural population from National Nature Reserve for *Cycas panzhihuaensis* in Sichuan, China (PZH), with seeds, coralloid roots, and rhizosphere soils collected on September 10th, 2016. Specifically, ten health *C*. *panzhihuaensis* individuals from KM and seven from PZH were chosen for sampling. Four samples per sample type were collected. Disposable gloves were worn and changed each time during different tissue type sampling.

Each sample was treated separately and stored at 4 °C, and DNA extraction was conducted as soon as possible after sampling. For soils, about 0.25 g of soil sample were added directly to the PowerBead Tubes provided by PowerSoil DNA Isolation Kit (MoBio) after removing plant debris and gravels. The mixtures were stored at 4 °C for DNA extraction. Before DNA extraction, plant debris and other impurities were picked out. Microbes on the surface of pollens, ovules, seeds and unfertilized seeds, or attaching to root epidermis, were eliminated to the highest degree possible according to the sterilization process conducted by Zheng et al. [26]. DNA extraction, PCR amplification, sequencing, and data analyses processes were all referred to in Zheng et al. [26].

During DNA extraction and sequencing process, one seed and one unfertilized seed sample from KM and one soil and one seed sample from PZH were failed with low sequence quality. Consequently, 32 samples from the two populations (22 from KM and ten from PZH) were used for further analyses.

### Statistical analysis

Except data analyzes conducted by Zheng et al. [26], additional statistical analyzes were also included, which were performed in R version 3.4.1. Before alpha diversity indices calculation, each sample was rarefied to the lowest sequencing depth (8611 for 16S rRNA sequences, 6081 for ITS sequences) in each library to minimize the error caused by sampling efforts across different compartments. The inverse Simpson diversity index [86], the OTU richness, and the Pielou’s evenness index [87] were calculated with 999 permutations. Normal distribution of the data was checked with the Shapiro-Wilk test and homogeneity of variances was analyzed using the Levene test in R with the ‘car’ package. Analysis of variance (ANOVA) was performed to verify whether significant difference of microbial diversity existed among sample types. Further, multiple pairwise comparisons between the means of groups at 95% family-wise confidence level were conducted based on the Tukey honest significant differences test (Tukey HSD).

To compare the composition of identified community members within different compartments and identify main factors driving community composition, the Bray-Curtis dissimilarity matrices were developed with the sequence normalized to 8611 per sample for 16S rRNA sequences and 6081 for ITS sequences. Principal component analyses (PCA), hierarchical cluster analyses (HCA), as well as heatmap analyses of microbial community structure were performed based on the Bray-Curtis dissimilarity matrix on the website METAGENAssist with the ‘Pearson’ distance measure and the ‘ward’ clustering algorithm [88]. To statistically support the above-mentioned visual clustering results of microbial community composition, PERMANOVA and pairwise comparison were conducted using the ‘adonis’ and ‘pairwise.adonis’ functions with the ‘bary’ method and 10,000 iterations by the ‘vegan’ package in R. By using the multipatt function of the ‘indicspecies’ package in R, indicator species analysis was carried out [89]. Before indicator species calculation, sequences were rarefied as in alpha diversity analysis, and the full family matrices were retrieved.

To have a comprehensive impression of the microbial community differentiation among different compartments, the core microbiome of each compartment (seeds, unfertilized seeds, ovules, pollens, roots and soils for KM; seeds, roots, and soils for PZH) was identified and compared. Firstly, the top ten families were retrieved for each compartment. Then, duplicate families among sample types were removed. ANOVA analysis was conducted to test the influence of different compartments on the microbial relative abundance. Additionally, Venn diagrams were drawn displaying the overlap of OTUs between different sample types by using BIOINFOGP [90] or InteractiVenn [91].

## Supplementary information

**Additional file 1.** Proportion of total bacterial (A) and fungal (B) reads assigned to taxonomic ranks.

**Additional file 2. **Core microbiomes in different compartments of *C. panzhihuaensis* revealed by 16S rRNA gene and ITS sequences, and their significant levels.

## Data Availability

The datasets used and/or analyzed during the current study are available from the corresponding author on reasonable request.
